# Double-sided liquid crystal metasurfaces for electrically and mechanically controlled broadband visible anomalous refraction

**DOI:** 10.1515/nanoph-2022-0091

**Published:** 2022-05-24

**Authors:** Maxim V. Gorkunov, Alena V. Mamonova, Irina V. Kasyanova, Alexander A. Ezhov, Vladimir V. Artemov, Ivan V. Simdyankin, Artur R. Geivandov

**Affiliations:** Shubnikov Institute of Crystallography, Federal Scientific Research Centre “Crystallography and Photonics”, Russian Academy of Sciences, 119333 Moscow, Russia; National Research Nuclear University MEPhI (Moscow Engineering Physics Institute), 115409 Moscow, Russia; Faculty of Physics, Lomonosov Moscow State University, 119991 Moscow, Russia; Topchiev Institute of Petrochemical Synthesis, Russian Academy of Science, 119991 Moscow, Russia

**Keywords:** anomalous refraction, electro-optical switching, mechanical tunability, metasurfaces, nematic liquid crystal

## Abstract

Liquid crystals self-assemble on nanopatterned alignment layers into purely soft matter metasurfaces sensitive to external stimuli and imparting tailored spatial modulations to transmitted light wavefronts. Upon fine optimization, they are capable of efficient light deflection by virtue of anomalous refraction into a dominating transmission diffraction order. To expand the spectral range and acquire additional functionality, we put forward the double-sided metasurface design based on the liquid crystal alignment by a pair of complementing patterned substrates. We numerically optimize, fabricate, and experimentally characterize metasurfaces refracting red light with an efficiency of up to 70% and sustaining the efficiency above 50% in a broad range of visible wavelengths exceeding 500 nm. We verify that the refraction is reversibly switched in less than 10 ms by voltages of a few volts. We also report on a remarkable mechanical reconfigurability, as micrometer-scale relative substrate shift flips the refraction direction.

## 1 Introduction

As optical metasurfaces evolve from an intriguing design concept [1] to the key part of innovative consumer devices [2], tunable configurations allowing for intelligent local control of electromagnetic properties receive growing attention [3]. Very different types of tunability have been proposed and studied, such as mechanical [4], optical (by powerful light) [5], or thermal [6]; among them, low-voltage electrical control is technically convenient and promises easy integration with existing electronic components.

Liquid crystals (LCs) are very well explored optical materials combining fluidity with optical anisotropy, stable upon ambient conditions, and highly sensitive to electric fields [7]. Since the first ideas of merging metastructures with LCs were put forward a decade ago [8–12], very different electrically tunable combinations of LCs with metallic metasurfaces [13–17] and dielectric metasurfaces [18–28] have been explored. Resonant optical properties of a metasurface can be controlled by realigning nanoscale LC volumes adjacent to the meta-atoms. In comparison with conventional LC display cells, one can drastically enhance the switching speed and sensitivity as well as significantly reduce the LC-layer thickness facilitating the scaling of the pixel size down to micrometers. On the other hand, one yet has to master predictable and reproducible LC alignment on such small scales [26–28].

In parallel, the development of photonic LC elements for nondisplay applications have shown that one can build very useful devices by employing LC self-assembling in regularly modulated structures stabilized by inhomogeneous aligning action of substrates [29–36]. As long as the latter are prepared by photopatterning of photosensitive alignment layers, the characteristic feature size of stabilized LC structures is bound to remain comparable with light wavelength. More recently, it has been found that one can establish similar periodic LC modulations upon polymer layers patterned by a focused ion beam (FIB) [37]. As the characteristic size of FIB patterns can be set well below the visible light wavelength, broad prospects have been opened for creating versatile LC structures of fine subwavelength design – LC-metasurfaces [38]. The latter have demonstrated efficient diffraction [39], deflection [40] and focusing [41] of visible light.

Controlled deflection of light beams is a key metasurface function required for meta-lensing and meta-holography, LiDAR and 3D depth sensing applications. It is conveniently realized as anomalous reflection and refraction performed by diffracting metastructures optimized to produce periodic saw-tooth-shaped phase retardation and to reroute the incoming light into a particular dominant outgoing diffraction order [42]. LC-metasurfaces stabilized by superperiodic alignment patterns optimized along this strategy have demonstrated the deflection of normally incident linearly polarized blue light into a particular oblique direction with 60% efficiency [40]. Importantly, applying alternating voltage of a few volts amplitude across LC layers can reversibly erase the metasurfaces and produce a several millisecond fast and infinitely repeatable switching between diffracting and transmitting states [39].

In this paper, we further advance the concept of light deflecting LC-metasurfaces by introducing their double-sided design. The main idea is to control the LC alignment by both LC-cell substrates, in order to substantially increase the range of phase retardation accessible by modulating reasonably thin LC layers. As described in Section 2, we develop the corresponding numerical optimization routine yielding superperiodic patterns for each substrate. Sections 3.1 and 3.2 demonstrate how imprinting such patterns on polymer alignment layers and assembling the corresponding LC cells indeed promotes the formation of LC metasurfaces which refract green, yellow, and red light into +1 transmission diffraction order with up to 60–70% efficiency. In Section 3.3 we account on the LC-metasurface electro-optics and, in particular, the switching dynamics. As discussed in Section 3.4, the double-sided design also enables remarkably sensitive mechanical reconfigurability by micrometer-scale relative shifts of the substrates. Finally, we present in Section 4 the main conclusions and outlook. Technical details of our theoretical and experimental methods are accounted in Section 5.

## 2 Design and optimization

### 2.1 Main idea

**Figure 1:**
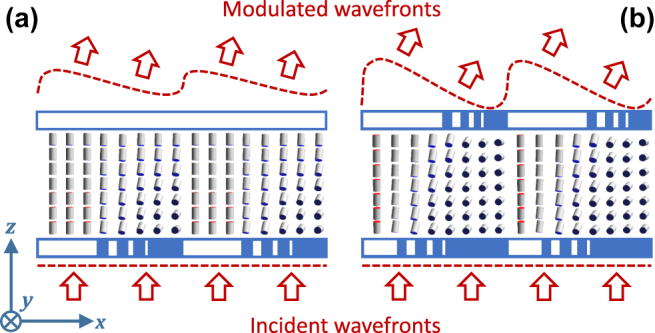
Sketches of anomalous light refraction by LC-metasurfaces self-assembling upon one patterned substrate (a) and between a pair of complementing patterned substrates (b). Substrate areas promoting vertical LC alignment are depicted in white.

The principle of anomalous light refraction by LC-metasurfaces is schematically shown in Figure 1. While the FIB patterned areas of alignment layers support the vertical (along the *z*-axis) LC director orientation, the pristine areas of rubbed polyimide align it in-plane along the rubbing direction (the *y*-axis). The goal is to establish a gradual variation of the LC director within each period by exploiting its orientational elasticity which smoothens the deformations as the LC is subjected to discontinuous binary boundary conditions.

When a single patterned substrate is used as in Ref. [40], the opposite one aligns the LC homogeneously vertically, see Figure 1(a), and the LC orientation is vertical at the beginning of each period and hybrid (varying from planar to vertical) at its end. When both substrates are patterned in a complementary manner, as in Figure 1(b), the LC alignment varies from vertical to planar. In both cases, the middle areas of each period are patterned with finer stripes to establish smooth transitions between the corresponding extreme cases.

As the light polarized linearly along the *y*-axis propagates through such LC layers, its optical path (OP) acquires saw-tooth-like periodic modulation. Qualitatively, in the areas with vertical LC director alignment, the light experiences lower ordinary LC refractive index *n*_o_ and travels faster. In the areas with planar or hybrid orientation, the larger extraordinary LC refractive index *n*_e_ also affects the light and slows it down. For efficient anomalous refraction, the amplitude of the OP modulation has to reach at least one wavelength *λ*.

For the single-sided design, the maximum accessible OP difference can be estimated as *δ* = Δ*nd*/2 [37, 43], where Δ*n* = *n*_e_ − *n*_o_ is the LC birefringence, and *d* is the thickness of the LC layer. It has been shown that a layer of E7 nematic LC mixture of convenient thickness about 4 μm can self-assemble into a single-sided metasurface performing reasonably efficient anomalous refraction of blue light in a 400–450 nm wavelength range [40]. To cover the broader visible spectrum, one can double the thickness *d*, which, however, would inevitably slow down the electro-optical switching by at least 4 times, worsen the LC alignment quality and promote defect formation. Using the double-sided design we overcome all such potential disadvantages as it allows modulating the OP with a twice larger amplitude *δ* = Δ*nd* by similarly thin LC layers which then deflect light in the whole visible range.

### 2.2 Numerical optimization

When subjected to aligning action of a patterned surface, the LC attains a fine balance between the abruptly modulated anchoring conditions and the anisotropic LC elasticity tending to smoothen the orientational deformations. The resulting equilibrium inhomogeneous states are characterized by complex spatial distributions of the LC director. The optical properties of such LC layers are rather nontrivial, as the light propagates through a uniaxial material which principal axis follows these complex LC director distributions.

Previously [40], we have developed a semi-analytical model accounting for the LC elasticity in terms of the single-constant approximation and also reducing the optical performance of a modulated LC layer to inhomogeneous OP accumulated by light propagating through it. However attractive such model is in terms of reducing the computational time, our preliminary estimates have shown its inaccuracy when applied to double-sided LC-metasurfaces. Therefore, we develop a much more elaborate numerical model using COMSOL Multiphysics, which obtains the equilibrium LC states with all its material properties precisely taken into account and also accurately solves the Maxwell equations for the light propagating through such complex states. The most important technical details are enclosed in Methods Section 5.1.

**Figure 2:**
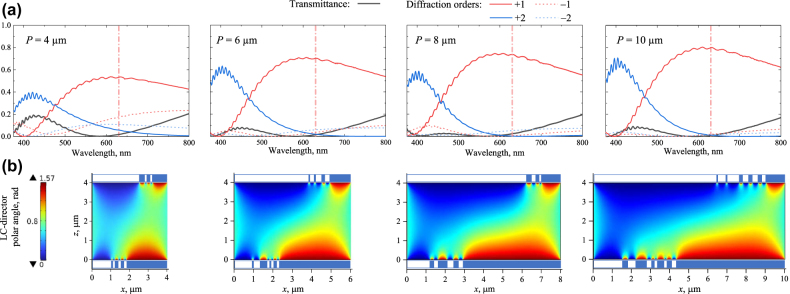
Double-sided LC-metasurfaces numerically optimized for maximum efficient anomalous refraction of red light into +1 diffraction order. (a) Spectra of transmittance and efficiencies of first diffraction orders (see the legend on the top) numerically evaluated for metasurfaces of the given periods *P*. Vertical dash-dot lines indicate the 630 nm wavelength taken for optimization. (b) Colormaps of the LC director polar angle distribution within single periods of the corresponding metasurfaces. The binary substrate aligning action is visualized as in Figure 1.

To remain within the current fabrication capabilities, we take four illustrative periods of 4, 6, 8, and 10 μm, set the LC layer thickness to 4 μm, and perform the optimization by varying the positions and widths of the stripes patterned on both substrates with a 100 nm resolution in order to achieve the maximum possible efficiency of +1 order diffraction of light with a wavelength of 630 nm. The results of such optimization are shown in Figure 2(a), where we present the spectra of the forward transmittance and the efficiencies of the strongest diffraction orders. The spectra vividly illustrate the potential advantage of the double-sided design as it promises achieving 60–70% efficient diffraction of linearly polarized red light and, for the metasurfaces of a period of 6 μm and larger; this efficiency stays above 50% in a broad wavelength range above 500 nm.

Such efficient refraction is based on the LC self-assembling into complex structures characterized by the director polar angle distributions shown in Figure 2(b). Note that for all the periods, the optimal distributions obey the same general principle schematically shown in Figure 1(b): first (from the left), the bottom substrate induces gradual reorientation of the adjacent LC from vertical to planar, which is then followed by a similar reorientation at the top substrate. At the same time, we see that in realistic simulations, the LC elasticity prevents it from aligning strictly in-plane in the right part of the period.

## 3 Experimental LC-metasurfaces

### 3.1 LC-cell preparation and adjustment

**Figure 3:**
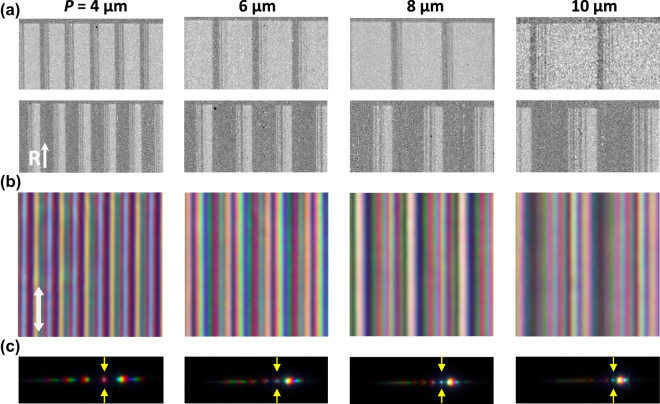
Experimental double-sided LC-metasurfaces self-assembling between FIB-patterned substrates. (a) SEM-images of fragments of pairs of substrates covered with polymer alignment layers rubbed in the direction of R-arrow and patterned by FIB with superperiodic stripes according to the optimized designs with the periods *P* indicated on the top. (b) POM-images in the light polarized along the stripes of LC metasurfaces self-assembled between the corresponding patterned substrates. (c) BFP-images of the LC metasurfaces with the spots of forward transmitted light indicated by yellow arrows.

The optimized patterns of superperiodic parallel stripes are imprinted by an FIB on polyimide alignment layers along the rubbing direction as described in Methods Section 5.2 yielding arrays of 350 × 350 μm^2^ square areas processed on the top and bottom substrates. Typical fragments of the edges of patterns with the periods 4, 6, 8, and 10 μm imprinted on both substrates are shown in Figure 3(a) as registered in the same scanning electron microscope (SEM) used for patterning. The surface areas subjected to FIB look brighter than the pristine ones. Note that according to the recent microscopic investigations [44], the FIB does not remove the polyimide film, but rather performs local chemical modification. The brighter shades of such areas arise due to a local modification of the ITO sublayer by the FIB and Ga implantation.

The assembling and coarse adjustment of the substrates is performed under a polarizing optical microscope (POM) and we generally observe reliable self-assembling of stable LC textures of an excellent quality exemplified in Figure 3(b). At the same time, we confirm that, as expected, the diffractive properties of the double-sided metasurfaces strongly vary as we perform their micrometer-precise relative adjustment using the technique described in Methods Section 5.3. At this stage, the metasurface images in the back focal plane (BFP) of an optical microscope, see Figure 3(c), are used for the guidance. As the metasurface designs were optimized for the maximum red light refraction, the fine relative adjustment of the substrates is performed to achieve a maximum strong +1 diffraction order seen in filtered red light.

### 3.2 Diffraction properties

**Figure 4:**
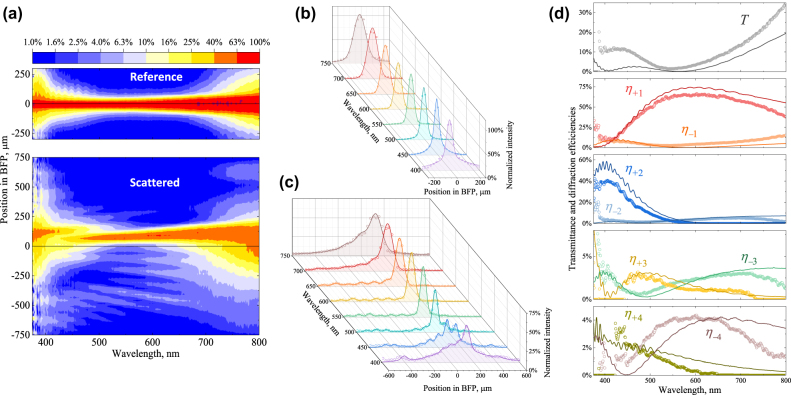
Extracting diffraction properties from the BFP-spectrometry data performed for the 8 μm periodic metasurface. (a) Intensity colormaps measured for the reference light transmitted by the homogeneous LC cell background and for the light scattered by the metasurface. Exemplary cross sections of the reference (b) and scattered (c) intensity colormaps with the measured intensity values shown by the circles and the lines displaying their analytical fits by Eqs. (2) and (1) in (b) and (c) respectively. Extracted spectra of transmittance and diffraction efficiencies are shown by circles in (d) compared to those predicted by numerical simulations (solid lines).

Although the BFP images contain valuable qualitative information on the relative strength of different diffraction orders, the unknown spectral sensitivity of the digital photo camera sensors does not allow us to obtain quantitatively correct spectra of diffraction efficiencies. For this purpose, we develop a specific BFP-spectrometry technique described in Methods Section 5.4. It relies on registering the light arriving at a specific position on the BFP and analyzing its spectral content with a fiber optic spectrometer. At the output, the technique yields the maps of intensity in the axes of position in the BFP and the wavelength, as exemplified in Figure 4(a). Note that further correct extraction of the diffraction efficiencies requires also precise knowledge of the reference light beam passing through a homogeneous LC-cell area.

For the known spatial profile of the reference beam intensity in the BFP at each wavelength, *I*_0_(*a*, *λ*), we assume the scattered light intensity map to be a superposition of spatially shifted reference beam profiles:

(1) $$I\left(a,\lambda \right)=\underset{n}{\sum }\eta_{n}\left(\lambda \right)I_{0}\left(a-a_{n},\lambda \right),$$

where *η*_*n*_(*λ*) are the diffraction efficiencies characterizing the relative part of the transmitted energy deflected into the corresponding order (*η*_0_ = *T* characterizes the forward transmission). The positions of diffraction maxima are determined by Bragg’s law: *a*_*n*_ = *a*_0_ + *nλH*/*P*, where *a*_0_ is the position of maximum of the forward transmitted beam, *P* is the metasurface period, and the distance *H* is a single constant characterizing the experimental setup. We determine the latter to provide the best coincidence of all *a*_*n*_ with the observed intensity maximums.

As the experimental data are provided on noncoincident grids of points in the (*aλ*) plane, the fitting of the experimental intensity by Eq. (1) is much simplified by an analytical approximation of the experimental profiles *I*_0_(*a*, *λ*). We find that a simple dependence

(2) $$\begin{array}{ll} I_{0}\left(a,\lambda \right) & =A\;\exp\left(-\frac{|a|+a}{2D_{1}}-\frac{|a|-a}{2D_{2}}\right)\\ & \;+B\;\exp\left(-\frac{a^{2}}{D_{3}^{2}}\right), \end{array}$$

with the parameters *A*, *B*, and *D*_*i*_ obtained by fitting *I*_0_(*a*, *λ*) at each wavelength, nicely reproduces the measured reference beam profiles, see Figure 4(b). Next, fitting the observed scattered intensity profiles appears to be also notably accurate as exemplified in Figure 4(c).

A comparison of the extracted spectra of efficiencies in Figure 4(d) with those predicted by the COMSOL Multiphysics simulations (same as in Figure 2) shows reasonably accurate quantitative agreement for the first 9 diffraction orders. We attribute somewhat lower peaks of experimental efficiencies to the light scattering on LC alignment imperfections.

**Figure 5:**
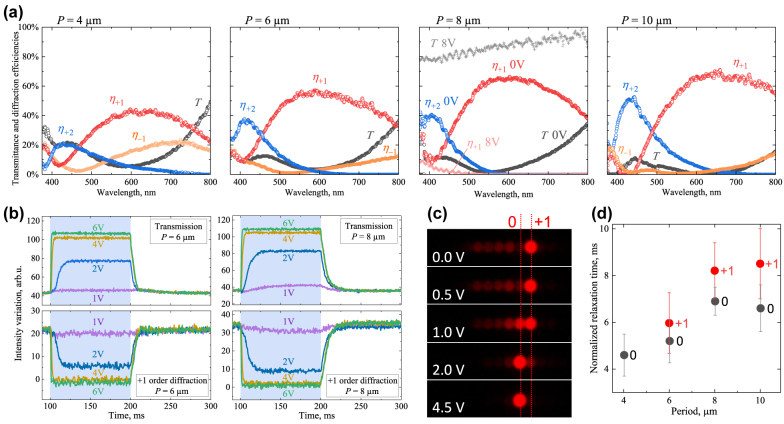
Diffraction properties of double-sided LC-metasurfaces and their electro-optical switching. (a) Spectra of transmittance and efficiencies of strongest diffraction orders of the metasurfaces of periods indicated on the top. Comparison with the spectra upon alternating voltage of a 8 V amplitude is shown for the 8 μm periodic metasurface. (b) Dynamics of variation of the intensity of light transmitted and diffracted into +1 order by the 6 μm and 8 μm periodic metasurfaces driven by voltages of the indicated amplitudes. Time intervals when the voltage is applied are shown by the light-blue background. (c) Variation of the optical BFP-images in red light of the 8 μm periodic metasurface upon applying the voltage of indicated amplitudes with the positions of the spots corresponding to 0 and +1 diffraction orders connected with dotted lines to guide the eye. (d) Combined times of normalized relaxation of 0 and +1 diffraction orders of all metasurfaces after the voltage is switched off. The diffraction orders are indicated directly at the dots, while the error bars show the ranges of relaxation time exhibited by metasurfaces switched by different voltage.

Applying this extraction procedure to the intensity maps measured by the BFP spectrometry of all fabricated metasurfaces, we obtain the efficiency spectra shown in Figure 5(a) which fully confirm the main expected advantage of the double-sided design. The LC metasurfaces do refract the red light with up to 60–70% efficiency, and also are notably broadband: those of periods of 6 μm and larger also sustain the +1 order efficiency higher than 50% for the wavelengths exceeding 500 nm.

### 3.3 Electro-optical switching

Similarly to the single-sided LC-metasurfaces, the double-sided ones are also notably susceptible to low voltages applied across the LC layer to the transparent ITO electrodes. Due to the positive LC dielectric anisotropy, the induced electric field promotes the vertical LC alignment throughout the whole cell volume, which suppresses the LC orientation modulations, and reroutes the diffracted light into the forward direction.

The experimental technique capable of resolving the switching dynamics with submillisecond resolution is outlined in Methods Section 5.5 and it is essentially similar to the previously used one [40]. As is conventional for the studies of LC systems, square pulses of alternating voltage of a frequency 1 kHz are used to avoid the cell heating and free charge accumulation at the electrodes.

Resolving the dynamics of switching of forward transmission (0 order) and +1 order diffraction, we obtain the characteristic behavior exemplified for the 6 μm and 8 μm periodic metasurfaces in Figure 5(b). The dynamics is qualitatively similar to that of the switching of single-sided metasurfaces [39, 40]: while the voltage-driven switching-on becomes millisecond-fast for strong enough voltage amplitudes, the switching-off is slower as it is governed by the LC viscosity, elasticity and the submicrometer-scale deformations induced by the substrates.

Considering the BFP images under the voltage, we do not observe substantial difference between the electro-optical behavior of metasurfaces of different periodicity. As is exemplified in Figure 5(c) by the set of BFP images in red light obtained for the 8 μm periodic metasurface upon different applied voltages, even a voltage of 1 V amplitude considerably alters the regime of red light diffraction and 0 and +1 orders become comparably strong. Further increasing the voltage amplitude to 4–5 V eliminates the red light diffraction, while the full suppression of diffraction in the whole visible range occurs at the voltages of 8–9 V amplitudes. The latter regime is illustrated by the additional transmission and diffraction spectra of the 8 μm periodic metasurface upon 8 V voltage also shown in Figure 5(a).

To reveal the general trends of relaxation dynamics, we normalize the optical power variation by the initial and final values and evaluate the normalized relaxation times during which the signal alters by *e* times for the metasurfaces of all periods relaxing after different voltages are switched off. Similarly to the previously studied single-sided LC-metasurfaces [39, 40], the normalized relaxation dynamics is weakly dependent on the voltage. We combine all acquired data on a single diagram in Figure 5(d), where the error bars show the ranges of the relaxation times manifested for different voltages. In all cases, the metasurfaces substantially relax within the first 10 ms and faster. The obvious general feature is that the relaxation slows down as the period increases. In this respect, the double-sided LC metasurfaces differ from their single-sided counterparts which relaxation speed is almost independent of the periodicity [40]. We attribute this peculiar difference to the fact that in the double-sided design, a part of the period is occupied by the LC aligned close to planar by the opposite substrates, see Figure 1(b). Such planar–planar configurations are known to possess very specific switching features and can even exhibit a Fredericksz transition-like threshold [7].

### 3.4 Mechanical reconfigurability

**Figure 6:**
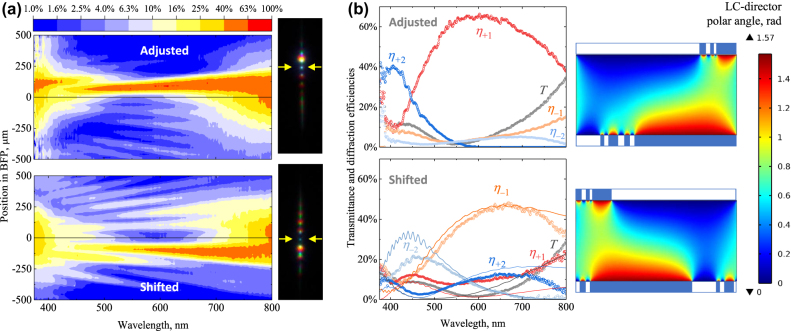
Mechanical tuning of the 8 μm periodic double-sided LC-metasurface. (a) Intensity colormaps obtained by BFP spectrometry when the substrates are properly adjusted and shifted to yield the maximum intense spots of +1 and −1 diffraction orders correspondingly. In the BFP images shown on the right the 0-order spots of forward transmission are indicated by yellow arrows. (b) Spectra of transmittance and diffraction efficiencies extracted from the intensity colormaps (circles) with the best fit of the shifted configuration obtained by COMSOL simulations (solid lines) assuming a 4 μm relative shift of the substrates. On the right: comparison of the corresponding distributions of the polar angle of LC director within one metasurface period when the substrates are exactly adjusted (same as in Figure 2(a)) and shifted by 4 μm with the coordinate origin chosen for better comparison. The binary substrate aligning action is visualized as in Figure 1.

In the course of finer adjustment of patterned LC-cell substrates, we encounter a peculiar specific feature of double-sided metasurfaces. While the proper adjustment produces the transmission and diffraction spectra fully in-line with theoretical expectations, it is also possible to enhance the red light refraction into the opposite −1 order by shifting the substrates by a fraction of metasurface period. A typical example is shown in Figure 6 for the same 8 μm periodic metasurface which behavior upon proper adjustment is analyzed in Figure 4. We clearly see on the intensity maps in Figure 6(a) as well as on the corresponding extracted spectra in Figure 6(b) that the efficiency of red light diffraction into −1 order can reach remarkably high values of 45%, while other orders remain profoundly suppressed in this part of the spectrum.

As the current substrate adjustment setup is not suitable for quantifying micrometer-scale differences in the relative positioning, we perform additional simulations in COMSOL Multiphysics for the metasurfaces self-assembling between differently shifted substrates. Comparing the spectra of simulated and measured efficiencies, we conclude that assuming the relative shift by 4 μm one can relatively well reproduce the experiment. In particular, this explains the −1 diffraction order efficiency maximum exceeding 45% around a wavelength of 670 nm, see Figure 6(b). In the simulations, we also observe a remarkable sensitivity of the optical properties to the relative substrate displacements as small as 100–200 nm. For example, taking a slightly different relative substrate shift of 4.2 μm, results in −1 diffraction order efficiency reaching its maximum at a 620 nm wavelength. Experimental proof of such intriguing possibilities is, however, beyond the capabilities of the current adjustment device and will require upgrading from mechanical micrometer screws to more precise piezo actuators.

The numerically obtained spatial distributions of the LC director polar angle also clarify the nature of such remarkable mechanical tunability. Plotting the distributions within one metasurface period for specifically chosen coordinate origin, as in Figure 6(b), we notice that the shapes of vertically aligned (blue color) and planarly aligned (red color) parts of the LC cell are similar up to a horizontal flipping. Note that the similarity is only qualitative, as, for instance, the shifted arrangement lacks a broader part of planar LC alignment by the opposing substrates. As a result, the peak of the refraction efficiency is somewhat lower.

## 4 Conclusions

Introduction of the double-sided design of LC-metasurfaces allowed expanding their operating range onto the whole visible spectrum and simultaneously sustain the LC alignment quality and switching speed. While analogous single-sided metasurfaces were capable of efficient deflection of blue light [40], now it is possible to refract red, yellow and green light with similar and even higher efficiency. The refraction can be conveniently switched off within a millisecond by applying an AC voltage of an 8 V amplitude. The relaxation back to the refracting state takes somewhat longer, but its main part occurs within less than 10 ms.

In addition, we encountered a very peculiar possibility to reconfigure the double-sided metasurfaces by micrometer-scale substrate displacements. Such mechanical (structural) tuning was previously realized with multilayer metasurfaces in the microwave range [4], where it relied on modifying the near-field electromagnetic coupling between parallel metalayers. In the LC-metasurfaces, a similar effect is more elaborate, as it involves the LC realignment by the substrates, which, in turn, alters the optical properties. However, the practical prospects remain similarly intriguing especially as the necessary mechanical shift of the order of several micrometers can be produced much easier and faster.

Finally, the studied designs were optimized for an operation with exactly adjusted substrates without voltage. It is very interesting to analyze if the double-sided LC-metasurfaces can be optimized for efficient operation in several states (controlled by weaker voltages or substrate shifts). We will address these questions in our future works.

## 5 Methods

### 5.1 Simulations

Numerical simulation and optimization of double-sided LC metasurfaces is performed using COMSOL Multiphysics in 2D mode. For a given configuration of stripes on both substrates, we assume rigid LC surface anchoring with the binary (0 or *π*/2) values of the director polar angle at the corresponding stripes on the substrates. Using more sophisticated but presumably also more realistic soft boundaries would require introducing a number of additional phenomenological parameters characterizing the anchoring strength on both patterned and pristine polyimide areas. As the simpler rigid conditions were previously sufficient for reproducing the experimental properties of single sided LC-metasurfaces [39, 40] we employ them here as well and conclude on their appropriateness on the basis of the achieved agreement between simulations and experiments.

The continuous LC deformation problem is solved in Weak Form PDE module with the elastic coefficients of the E7 nematic mixture equal to [45]: *K*_11_ = 11.1 pN, *K*_22_ = 6.5 pN and *K*_33_ = 17.1 pN, and with the LC layer thickness set to 4 μm.

The optical performance is evaluated by an accurate solution of the Maxwell equations in EWFD module of COMSOL Multiphysics taking into account the actual LC cell structure including the glass substrates and the 150 nm thick ITO electrodes. All optical material properties, as previously [40], are adopted from the available independent experimental data: the ITO refractive index is taken from the COMSOL material library: In_2_O_3_-SnO_2_ (König et al. 2014: *n*, *k* 0.25–1.0 μm) [46], while the principal permittivity values of the E7 Merck nematic mixture are set in terms of Lorentzian model fitting their experimental dispersion [47]. In a same way as in the optical measurements, the transmittance and diffraction efficiencies are normalized by the forward transmittance of a separately simulated homogeneous area of the LC-cell subjected to identical illumination.

For the optimization, the *x*-coordinates of the edges of aligning stripes, to be imprinted by FIB, are used as variables. During the optimization, the configurations with merged or overlapping stripes are excluded by limiting the gaps between them to be at least 100 nm wide. The initial stripe configurations are taken as obtained by the semi-analytical approximate model [40]. In spite of its inaccuracy for double-sided LC-metasurfaces, the model yields starting points reasonably close to the optimum. The efficiency of +1 diffraction order at a red laser wavelength of 630 nm is taken as objective function. The tolerance value which determines the proximity to the stationary solution is taken equal to 0.01, and the maximum number of model evaluations is limited to 1000, except for the largest period of 10 μm, for which it is reduced to 500, to avoid much longer computation times. The optimal number of vertically aligning stripes in each unit cell appears to be not necessarily equal on the opposing substrates and tends to grow with the increase of metasurface periodicity. The particular coordinates of the stripe edges are listed in Table 1.

**Table 1:** Optimal configurations of vertically aligning stripes on both substrates of LC-metasurfaces of different periods. The *x*-coordinates of stripe edges are given in pairs as (begin, end). All values are expressed in micrometers.

Period	Top substrate	Bottom substrate
4	(0.0,2.5)(2.8,2.9)	(0.0,1.0)(1.1,1.2)
	(3.1,3.2)	(1.4,1.5)(1.7,1.8)
6	(0.0,3.8)(3.9,4.1)	(0.0,0.9)(1.0,1.3)
	(4.2,4.5)(4.7,4.9)	(1.7,1.8)(1.9,2.0)
		(2.2,2.3)
8	(0.0,6.2)(6.5,6.7)	(0.0,1.2)(1.4,1.6)
	(6.9,7.0)	(2.1,2.4)(2.7,2.9)
10	(0.0,6.4)(6.5,6.9)	(0.0,1.5)(1.8,2.2)
	(7.0,7.6)(7.8,8.1)	(2.8,3.0)(3.2,3.3)
	(8.3,8.6)(8.8,9.0)	(3.7,3.8)(4.1,4.3)

For the optimized stripe configurations, the spectra of transmittance and diffraction efficiencies are obtained in a similar manner by EWFD module of COMSOL Multiphysics. The relative adjustment of stripes on both substrates is presumed to be exact for the plots in Figures 2(a) and 4(d). For Figure 6(b), the optical spectra corresponding to different relative shifts of the stripe patterns of the 8 μm periodic metasurface are evaluated, and the combination best fitting the experimental data is presented.

To assess the robustness of the optimal double-sided configurations with respect to inevitable deviations of the LC-cell gap, we perform additional simulations assuming that the substrates are exactly adjusted and separated by gaps of 3.5 μm and 4.5 μm. The numerical data demonstrate that even such significant changes of the gap result only in subtle spectral shifts of the maximum of +1 diffraction order efficiency by about 20 nm on the wavelength scale, while the target maximum value remains intact. Therefore, we conclude that optimal designs of the double-sided metasurfaces are sufficiently robust and proceed with their fabrication.

### 5.2 Substrate preparation

The initial homogeneously aligning substrates are prepared according to the established routine. Standard soda-lime display-quality glass plates carrying 150 nm thick transparent ITO electrodes are cleaned mechanically and by atmospheric plasma. Further, they are spin-coated with a 1% solution of polyamic acid in dimethylformamide and annealed at 250 °C for 1 h. Finally, a unidirectional rubbing with a soft cotton cloth is applied to establish uniform planar LC director alignment. The substrates include contacts soldered to the ITO electrodes allowing applying the voltage.

As previously [39, 40], we pattern the rubbed polyimide layers by mild irradiation of the FIB using an FEI Scios DualBeam electron microscope controlled by digital raster templates – stream files. The files are generated by a Matlab code according to the optimized designs and enable processing square areas of 3500 × 3500 pixel^2^ in size. We set the microscope magnification to ensure that a single pixel corresponds to a 0.1 × 0.1 μm^2^ area of the substrate surface and imprint the patterns by a beam of Ga^+^ ions of a current of 0.1 nA accelerated by a voltage of 30 kV with the dwell-time of 150 μs of each irradiated pixel.

### 5.3 LC-cell assembling and adjustment

A pair of patterned substrates is stacked and assembled in an LC-cell with the gap fixed with Sekisui Micropearl plastic spacers of 4 μm in diameter. Using the Fabri–Perot oscillations of the transmittance of empty cell we evaluate the actual gap to be close to 4 μm with an accuracy of ±0.1 μm. After that, the cell is filled with E7 (Merck) nematic LC in the isotropic phase.

Relative adjustment of the patterns on opposing substrates is performed using a custom made device [48] comprising two 3D-printed plastic frames equipped with Newport Vernier Micrometer SM-13 screws for fine adjustment. While one LC cell substrate is fixed in the bottom frame, the other is glued to the top frame and remains in contact with the screw ball heads: two screws at the long side and one at the short side. The design allows moving the top frame along the cell plane and, in addition, performing angular corrections using two screws on the longer side to match both the relative pattern position and orientation. To sustain the cell gap, the substrates are clamped together by a pair of neodymium magnets leant to the outer glass surfaces. The device allows placing the LC-cell on the table of an optical microscope and precisely adjusting the double-sided LC metasurfaces in the course of their optical characterization.

### 5.4 BFP spectrometry

**Figure 7:**
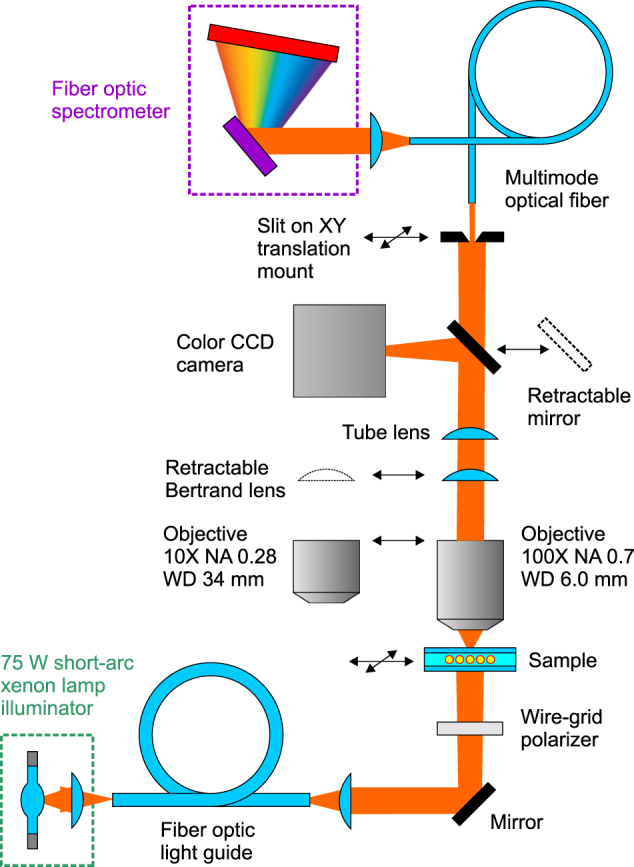
Schematic of experimental setup used for BFP spectrometry.

The measurements are carried out using a custom setup schematically depicted in Figure 7. It is based on Olympus BX53MTRF optical microscope and Ocean Optics QE Pro fiber optic spectrometer, and employs a short-arc xenon lamp of a power of 75 W as a light source. Standard microscope objectives are replaced with long working distance Mitutoyo Plan Apo Infinity Corrected Long WD 10X and 100X ones. The 10X objective is used for selecting appropriate LC-cell areas. The 100X objective is employed for the BFP microscopy and spectrometry as its field of view is less than 250 μm in diameter and allows collecting the light transmitted by the metasurfaces avoiding contribution from the uniform LC-cell background. The linear polarization of light incident on the samples is established by a wire-grid polarizer. Olympus DP26 CCD camera is used to monitor the substrate adjustment and to capture the images of the studied areas as well their images in the BFP.

To register the light arriving at specific different points in the BFP and to analyze its spectral content, the input end of the spectrometer fiber is attached to an XY translation mount equipped with custom-made stepper motor actuators controlled by an Arduino microprocessor board. To limit the signal acquisition area, a 50 μm wide slit oriented perpendicular to the diffraction orders is installed in the BFP of the assembly of Bertrand and tube lenses. The operation of spectrometer is controlled by OceanView software and a custom software programmed in the LabView environment under the general control of a script written in Windows PowerShell.

### 5.5 Switching dynamics measurement

The electro-optical measurements are performed at a custom setup based on Olympus CX31PF-5 microscope (built-in objective of a numerical aperture of 0.25) mounted with semiconductor laser diode (wavelength 445 nm) as a light source, with a linear wire-grid polarizer Moxtek UBB01A, a collimating lens of a focal length of 5 mm in front of the sample, a custom diaphragm, and a silicon photodiode for signal registration. The light is polarized along the pattern stripes and focused at the sample plane so that the beam spot size does not exceed the metasurface dimensions. The custom 3D-printed diaphragm in front of the photodiode has a moving part with a hole of 1 mm in diameter allowing separating and independently analyzing the forward transmission and +1 diffraction order. The voltage is applied to the LC-cell in the form of 100 ms long rectangular pulses filled with square waves of a frequency of 1 kHz. The generation of driving waveforms as well as the detected signal registration is performed in an external trigger mode using custom Physlab Virtual Instruments software.

## References

[1] Kildishev A. V., Boltasseva A., Shalaev V. M. (2013). Planar photonics with metasurfaces. *Science*.

[2] Chen W. T., Capasso F. (2021). Will flat optics appear in everyday life anytime soon?. Appl. Phys. Lett..

[3] Tsilipakos O., Tasolamprou A. C., Pitilakis A. (2020). Toward intelligent metasurfaces: the progress from globally tunable metasurfaces to software-defined metasurfaces with an embedded network of controllers. Adv. Opt. Mater..

[4] Lapine M., Powell D., Gorkunov M., Shadrivov I., Marqués R., Kivshar Y. (2009). Structural tunability in metamaterials. Appl. Phys. Lett..

[5] Shcherbakov M. R., Vabishchevich P. P., Shorokhov A. S. (2015). Ultrafast all-optical switching with magnetic resonances in nonlinear dielectric nanostructures. Nano Lett..

[6] Rahmani M., Xu L., Miroshnichenko A. E. (2017). Reversible thermal tuning of all-dielectric metasurfaces. Adv. Funct. Mater..

[7] Blinov L. M. (2011). Structure and Properties of Liquid Crystals.

[8] Kossyrev P. A., Yin A., Cloutier S. G. (2005). Electric field tuning of plasmonic response of nanodot array in liquid crystal matrix. Nano Lett..

[9] Gorkunov M. V., Osipov M. A. (2008). Tunability of wire-grid metamaterial immersed into nematic liquid crystal. J. Appl. Phys..

[10] Minovich A., Farnell J., Neshev D. N. (2012). Liquid crystal based nonlinear fishnet metamaterials. Appl. Phys. Lett..

[11] Decker M., Kremers C., Minovich A. (2013). Electro-optical switching by liquid-crystal controlled metasurfaces. Opt. Express.

[12] Buchnev O., Ou J. Y., Kaczmarek M., Zheludev N. I., Fedotov V. A. (2013). Electro-optical control in a plasmonic metamaterial hybridised with a liquid-crystal cell. Opt. Express.

[13] Buchnev O., Podoliak N., Kaczmarek M., Zheludev N. I., Fedotov V. A. (2015). Electrically controlled nanostructured metasurface loaded with liquid crystal: toward multifunctional photonic switch. Adv. Opt. Mater..

[14] Palto S. P., Barnik M. I., Kasyanova I. V. (2016). Plasmon electro-optic effect in a subwavelength metallic nanograting with a nematic liquid crystal. JETP Lett. (Engl. Transl.).

[15] Gorkunov M. V., Kasyanova I. V., Artemov V. V., Barnik M. I., Geivandov A. R., Palto S. P. (2017). Fast surface-plasmon-mediated electro-optics of a liquid crystal on a metal grating. Phys. Rev. Appl..

[16] Xie Z.-W., Yang J.-H., Vashistha V., Lee W., Chen K.-P. (2017). Liquid-crystal tunable color filters based on aluminum metasurfaces. Opt. Express.

[17] Wu J., Shen Ze., Ge S. (2020). Liquid crystal programmable metasurface for terahertz beam steering. Appl. Phys. Lett..

[18] Sautter J., Staude I., Decker M. (2015). Active tuning of all-dielectric metasurfaces. ACS Nano.

[19] Komar A., Fang Z., Bohn J. (2017). Electrically tunable all-dielectric optical metasurfaces based on liquid crystals. Appl. Phys. Lett..

[20] Komar A., Paniagua-Domínguez R., Miroshnichenko A. (2018). Dynamic beam switching by liquid crystal tunable dielectric metasurfaces. ACS Photonics.

[21] Zou C., Komar A., Fasold S. (2019). Electrically tunable transparent displays for visible light based on dielectric metasurfaces. ACS Photonics.

[22] Sun M., Xu X., Sun X. W. (2019). Efficient visible light modulation based on electrically tunable all dielectric metasurfaces embedded in thin-layer nematic liquid crystals. Sci. Rep..

[23] Li S.-Q., Xu X., Veetil R. M., Valuckas V., Paniagua-Domínguez R., Kuznetsov A. I. (2019). Phase-only transmissive spatial light modulator based on tunable dielectric metasurface. Science.

[24] Chung H., Miller O. D. (2020). Tunable metasurface inverse design for 80% switching efficiencies and 144° angular deflection. ACS Photonics.

[25] Rocco D., Carletti L., Caputo R., Finazzi M., Celebrano M., De Angelis C. (2020). Switching the second harmonic generation by a dielectric metasurface via tunable liquid crystal. Opt. Express.

[26] Lininger A., Zhu A. Y., Park J.-S. (2020). Optical properties of metasurfaces infiltrated with liquid crystals. Proc. Natl. Acad. Sci. Unit. States Am..

[27] Bosch M., Shcherbakov M. R., Won K., Lee H.-S., Kim Y., Shvets G. (2021). Electrically actuated varifocal lens based on liquid-crystal-embedded dielectric metasurfaces. Nano Lett..

[28] Dolan J. A., Cai H., Delalande L. (2021). Broadband liquid crystal tunable metasurfaces in the visible: liquid crystal inhomogeneities across the metasurface parameter space. ACS Photonics.

[29] Slussarenko S., Murauski A., Du T., Chigrinov V., Marrucci L., Santamato E. (2011). Tunable liquid crystal q-plates with arbitrary topological charge. Opt. Express.

[30] Wang X.-Q., Srivastava A. K., Chigrinov V. G., Kwok H.-S. (2013). Switchable fresnel lens based on micropatterned alignment. Opt. Lett..

[31] Kim J., Li Y., Miskiewicz M. N., Oh C., Kudenov M. W., Escuti M. J. (2015). Fabrication of ideal geometric-phase holograms with arbitrary wavefronts. Optica.

[32] Glazar N., Culbreath C., Li Y., Yokoyama H. (2015). Switchable liquid-crystal phase-shift mask for super-resolution photolithography based on pancharatnam–berry phase. Appl. Phys. Express.

[33] De Sio L., Roberts D. E., Liao Z. (2017). Beam shaping diffractive wave plates [invited]. Appl. Opt..

[34] Guo Y., Jiang M., Peng C. (2016). High-resolution and high-throughput plasmonic photopatterning of complex molecular orientations in liquid crystals. Adv. Mater..

[35] Ma L.-L., Wu S.-Bo., Hu W. (2019). Self-assembled asymmetric microlenses for four-dimensional visual imaging. ACS Nano.

[36] Yin K., Xiong J., He Z., Wu S.-T. (2020). Patterning liquid-crystal alignment for ultrathin flat optics. ACS Omega.

[37] Kasyanova I. V., Gorkunov M. V., Artemov V. V., Geivandov A. R., Mamonova A. V., Palto S. P. (2018). Liquid crystal metasurfaces on micropatterned polymer substrates. Opt. Express.

[38] Kasyanova I. V., Gorkunov M. V., Palto S. P. (2021). Liquid-crystal metasurfaces: self-assembly for versatile optical functionality. Europhys. Lett..

[39] Gorkunov M. V., Kasyanova I. V., Artemov V. V. (2020). Liquid-crystal metasurfaces self-assembled on focused ion beam patterned polymer layers: electro-optical control of light diffraction and transmission. ACS Appl. Mater. Interfaces.

[40] Gorkunov M. V., Kasyanova I. V., Artemov V. V. (2020). Superperiodic liquid-crystal metasurfaces for electrically controlled anomalous refraction. ACS Photonics.

[41] Palto S. P., Geivandov A. R., Kasyanova I. V., Simdyankin I. V., Artemov V. V., Gorkunov M. V. (2021). Liquid crystal microlenses based on binary surface alignment controlled by focused ion beam treatment. Opt. Lett..

[42] Yu N., Genevet P., Kats M. A. (2011). Light propagation with phase discontinuities: generalized laws of reflection and refraction. Science.

[43] Drolet J.-J. P., Patel J. S., Haritos K. G., Xu W., Scherer A., Psaltis D. (1995). Hybrid-aligned nematic liquid-crystal modulators fabricated on VLSI circuits. Opt. Lett..

[44] Artemov V. V., Khmelinin D. N., Mamonova A. V., Gorkunov M. V., Ezhov A. A. (2021). Microscopic studies of alignment layers processed by a focused ion beam for the creation of liquid crystal metasurfaces. Crystallogr. Rep..

[45] Pestov S., Vill V. (2018). Liquid crystals. *Springer Handbook of Materials Data*.

[46] Tkachenko V., Abbate G., Marino A. (2006). Nematic liquid crystal optical dispersion in the visible-near infrared range. Mol. Cryst. Liq. Cryst..

[47] Geivandov A. R., Kasyanova I. V. (2020). Device for alignment of microstructures on two substrates with micrometric precision. *Pribori i tekhnika eksperimenta*.

[48] König T. A. F., Ledin P. A., Kerszulis J. (2014). Electrically Tunable Plasmonic Behavior of Nanocube–Polymer Nanomaterials Induced by a Redox-Active Electrochromic Polymer. *ACS Nano*.

